# Vaccine Hesitancy: In Search of the Risk Communication Comfort Zone

**DOI:** 10.1371/currents.outbreaks.0561a011117a1d1f9596e24949e8690b

**Published:** 2017-03-03

**Authors:** Joshua Greenberg, Eve Dubé, Michelle Driedger

**Affiliations:** School of Journalism and Communication, Carleton University, Ottawa, Ontario, Canada; Institut National de Santé Publique du Québec, Québec, Canada; Centre de Recherche du CHU de Québec, Université Laval, Québec, Canada; Department of Community Health Sciences, Max Rady College of Medicine, University of Manitoba, Winnipeg, Manitoba, Canada

**Keywords:** anti-vaccination, health communication, immunization, MMR vaccine, public health, risk communication, vaccine hesitancy

## Abstract

**Introduction:**

This paper reports the findings of a national online survey to parents of children aged 5 and younger. The objectives of the study were to assess parental understanding of childhood immunizations, identify sources of information that they trust for vaccine-related content, assess where parents with young children stand on the key issues in the public debate about vaccination, and identify which risk communication messages are most effective for influencing the behaviours of vaccine hesitant parents.

**Methods:**

A total of 1,000 surveys (closed and open-ended questions) were administered in November 2015 using the Angus Reid Forum Panel, a key consumer panel consisting of approximately 150,000 Canadian adults aged 18 and older, spread across all geographic regions of Canada.

**Results:**

Approximately 92% of the Canadian parents surveyed consider vaccines safe and effective, and trust doctors and public health officials to provide timely and credible vaccine-related information. However, a concerning number of them either believe or are uncertain whether there is a link between vaccines and autism (28%), worry that vaccines might seriously harm their children (27%), or believe the pharmaceutical industry is behind the push for mandatory immunization (33%). Moreover, despite the common assumption that social media are becoming the go-to source of health news and information, most parents still rely on traditional media and official government websites for timely and credible information about vaccines and vaccine preventable diseases, particularly during community-based disease outbreaks. Finally, parents reported high levels of support for pro-vaccine messaging that has been demonstrated in previous research to have little to no positive impact on behaviour change, and may even be counterproductive.

**Discussion:**

The study’s results are highly relevant in a context where public health officials are expending significant resources to increase rates of childhood immunization and combat vaccine hesitancy. The data offer insight into where parents stand on the political and public debate about mandatory vaccination, what aspects of vaccine science remain uncertain to them, which media and institutional sources they use and trust to navigate the health information environment, how they look for information and whom they trust during periods of health emergency or crisis, and which communication strategies are considered most effective in persuading vaccine hesitant parents to immunize their children.

## Introduction

In March 2014, Toronto Public Health launched a media campaign to pressure the popular ABC daytime talk show, The View, to reverse its decision to hire outspoken celebrity, Jenny McCarthy, as one of its standing hosts. The concerns, outlined in a series of tweets, Facebook posts and news interviews, were that McCarthy’s well-known anti-vaccine activism would continue to undermine efforts by medical and public health authorities to increase vaccination uptake at a time when measles, whooping cough, chicken pox, mumps and other vaccine preventable diseases (VPD) have been making a comeback. McCarthy co-hosted The View for a year, and was eventually, and suddenly, released from the show in August 2015, reportedly due in part to her unpopular views about vaccines.

Anti-vaccine sentiment has emerged as a recurring topic of media attention alongside each new outbreak of vaccine-preventable disease. As the Toronto Public Health campaign illustrates, it has also become a focus of professional advocacy. While the number of Canadian parents who hold strident anti-vaccine beliefs and refuse to vaccinate their children is generally low (fewer than 3%), increasing numbers of parents (up to 35% in Canada) fall somewhere along a spectrum of beliefs and behaviours that we today call 'vaccine hesitant' (VH). Vaccine hesitant parents are a heterogeneous group who express worries about the risks and side effects of vaccines, including when and how frequently they should be administered. While some parents avoid vaccination altogether, others will agree to some vaccines but not all. Vaccine hesitant parents may also ultimately consent to have their kids vaccinated, but do so reluctantly because they remain uncertain about whether this is the correct course of action to take for their child's health 1.

We argue that the uncertainty associated with vaccine hesitancy represents a 'wicked' risk communication problem for public health officials. Wicked problems are by their nature difficult to define, and the solutions for addressing them are often uncertain 2. Although there is broad consensus among health scientists, medical professionals, policymakers and a majority of Canadians that childhood vaccination is a vital public health intervention, there is less agreement about how how public health officials can effectively communicate with parents to address their worries, beliefs and concerns about vaccines. Parents live within and have to navigate an increasingly complex, noisy mediascape in which conflicting claims about vaccines and VPDs compete for attention and thus shape their affective environment. As illustrated by the Toronto Public Health campaign, health officials are challenged to cut through this noise to make the most persuasive case possible that vaccines are both necessary for community protection and essential to saving lives.

In large part, this is a framing problem—how do we talk about vaccine hesitancy, to what do we attribute this phenomenon, and what solutions do we consider? As Scheufele^3^ argues, frames are both cognitive schemata (i.e., the stories and scripts we carry around in our heads) and elements of public discourse (i.e., manifest or latent patterns of media texts and public communication). Vaccine hesitancy is commonly framed in both media and public discourse as a knowledge-deficit problem—if only parents had access to accurate, scientifically verifiable information about the safety and efficacy of vaccines then surely they would embrace the miracle of immunization. It is also often framed as an example of irrational or misinformed thinking—if only people were not so easily tricked by junk science or the power of Big Pharma and celebrity culture then they would surely be less afraid of the risks and line their kids up for their shots.

Compelling as these accounts may be, neither is empirically tenable. Rather, as we know from recent research, vaccine hesitancy is driven by a complex configuration of issues, including, but not limited to, poor public health literacy, public perceptions of risk that are disproportionate to expert assessments, cultural values, access to large amounts of conflicting information, declining trust in experts, and a host of socio-demographic factors 4 ^,^ 5 ^,^ 6 ^,^ 7. How media, health advocates and others frame the problem of vaccine hesitancy is of course influenced by and informs these issues. And today, public health officials struggle with how best to respond to parental worries, concerns and vaccine refusal behaviours 8.

Our objectives for this study were to know more about perceptions of Canadian parents regarding vaccines and vaccine-preventable disease, to determine what information sources they use, and which institutions they trust. We also explored the responses of parents to common risk messages intended to persuade those who are vaccine hesitant to have their kids vaccinated.

## Methodology

To understand and inform strategies for addressing vaccine hesitancy we administered an online survey to 1,000 Canadians parents with young children (5 and younger) regarding immunization-related decision-making, and focusing specifically on the MMR vaccine. The survey instrument included 25 questions organized into 4 major categories: perceptions about vaccines and vaccination; views on the public debate about vaccines and vaccine-preventable disease; information seeking needs and practices, including media usage and trust in institutional sources; and communication strategies.

Participants were provided a 5-point Likert Scale (Strongly Agree, Agree, Disagree, Strongly Disagree, No Opinion/Don't Know) to respond to the majority of questions, particularly those relating to the 'Perceptions of Vaccination' and 'Public Debate' items. We also posed three open-ended questions to provide respondents with an opportunity to: (a) explain their vaccination choices; (b) describe where they get their health information; and (c) offer suggestions of other communication strategies that might persuade vaccine hesitant parents. We had two screening questions and a battery of brief questions designed to provide demographic profiles of our respondents. The survey took approximately 15-18 minutes to complete.

Research participants were recruited in December 2015 using the Angus Reid Online Forum panel, a key consumer panel consisting of approximately 150,000 Canadian adults aged 18 and older, spread across all geographic regions of Canada. The panel is benchmarked to known Census targets, such as age, region, income, and education, to ensure a representative sample of the Canadian population. Angus Reid's online panels are constituted by Canadians who register themselves as participants in surveys for compensation, such as cash, gift cards or vouchers. To be eligible, participants must complete a demographic profile of themselves so that a sample can be created which matches the targeted population of each study. In this case, our interest was to survey only parents with vaccine-aged children, thus the survey would have been distributed only to registered participants matching this profile. Participants are under no obligation to complete surveys once they have registered.

The participation rate for the study was 89 percent. We reached our target participation number of 1,000 respondents after distributing the survey to 1,121 participants who met our qualifying criteria.

## Results

*How many parents vaccinate, and what are their perceptions about vaccines?*

A majority of parents we surveyed, 92 percent, have had their children immunized with the MMR vaccine. Nearly half of the 6 percent of parents whose children had not been vaccinated reported that the age of their child was the primary factor (i.e., they were too young to receive the vaccine). In Canada, the vaccine schedule varies depending on where one lives in the country. In Ontario, children receive two doses, the first at aged 12 to 15 months, and a second dose (MMRV) at 18 months or anytime thereafter and before beginning school. Among the 3.9 percent of survey respondents who indicated that they have intentionally chosen to not vaccinate children, the major concerns given were fears about the possibility of serious adverse reactions (25 percent) and skepticism about vaccine effectiveness (11 percent).

There is a strong consensus within the international public health community (WHO, European Commission, NHS, CDC, Public Health Agency of Canada, American Academy of Paediatrics, etc.) that vaccines are safe, provide tremendous benefits to children's health, and produce relatively minor side effects. Yet, despite the strong record and scientific consensus about vaccine safety and efficacy, some parents remain skeptical, nervous and uncertain about whether vaccination is appropriate for their children. We were interested, first, to know whether parents have the same levels of understanding and confidence about vaccine safety and efficacy as medical professionals.

A majority of parents correctly answered a series of basic questions about vaccines and vaccination, although some questions elicited higher rates of incorrect and uncertain responses. More than 90 percent of parents agreed with the statement, “vaccines are safe and effective at preventing childhood illnesses” and 82 percent agreed that there is “clear consensus among medical experts that vaccines are safe.” Similarly, 83 percent also disagreed with the statement, “vaccination is less important than it used to be because we have eradicated most childhood diseases.”

Yet, our survey results also revealed some worrisome findings. First, that nearly 17 percent of parents consider vaccination "less important today than in the past" should be a source of concern given the resurgence of VPDs such as measles, mumps and whooping cough. It also reflects findings of other research showing rising rates of vaccine hesitancy attributed to beliefs about vaccine necessity 9. Most troubling, 14 percent of parents agreed with the statement, “vaccines can cause autism,” and an additional 14 percent expressed uncertainty about this statement. Second, although serious vaccine related injuries are rare, more than one-quarter of respondents agreed or were unsure about the statement, “there is a strong likelihood that the MMR vaccine will produce serious adverse reactions.” This indicates that even among parents who vaccinate, some of the most dangerous claims raised by the anti-vaccination movement—vaccines will injure your child and can cause autism—have achieved a concerning degree of resonance.

*Where do parents stand on the public debate about vaccines?*

Vaccine hesitancy has become a high profile news topic, as illustrated by the Toronto Public Health campaign described in the introduction to this paper. We wanted to understand where parents position themselves on the myriad issues that constitute this increasingly polarized, public debate.

Parent views on vaccines as a 'public issue' were more varied than they were on the knowledge of vaccine and vaccination questions. For example, the issue of mandatory vaccination was particularly contentious. Survey respondents were almost evenly split in agreement when asked, “should parents be able to choose whether their children are vaccinated?” Even though more than 90 percent of parents have had their kids vaccinated, 44 percent agreed that vaccination should still be a matter of parental choice (49 percent disagreed, and 7 percent were unsure). We also asked whether schools and daycare facilities should refuse children who are not vaccinated, except for those with medical exemptions. Approximately 65 percent of our respondents answered this question affirmatively, and 66 percent agreed with the statement, “parents who do not have their children immunized (except in cases involving medical exemptions) are irresponsible.” Furthermore, while a strong majority of parents have had their kids vaccinated, and many of them agree that there is a scientific consensus about vaccine safety and effectiveness, 33 percent agree with the statement, “drug companies are behind the government’s push for mandatory vaccination.” Here again, as with the responses to the autism link statement, a key piece of the anti-vaccine movement’s rhetorical artillery has struck a chord with parents.

*Where do parents get their health information?*

We asked parents an open-ended question: which media sources do you most use for health news and information? For the majority of respondents (57 percent), online news and information sources (e.g., Google, social media, websites, etc.) are their preferred source for health news and information, followed by television or radio (29 percent). A very small number of parents (5 percent) indicated that they most often use scientific sources, such as medical journals, to navigate the ever-changing health information landscape.

We also posed a series of situational questions designed to understand where parents would go first and most often for information and guidance if an outbreak of VPD were to pose a risk to their community. We expected social media sites such as Twitter or Facebook to be obvious top information sources given how many parents indicated that they use the Internet to keep abreast of health news and events, and because of the ways in which these platforms have become key sites of breaking news and collaborative news curation 10. However, this was not the case at all—only 14 percent of our respondents indicated that they would consult social media for breaking news and information if an outbreak of VPD were to threaten their communities. Instead, the website of major news organizations was the overwhelming top choice (33 percent), followed by official government websites (24 percent) and television stations (19 percent). These patterns also roughly translated to the preferred information sources that parents would consult most often.

*Which institutions and sources do parents trust?*

Past research is clear that the most trusted source of vaccine related information are family physicians and other medical professionals 11. The growth of the anti-vaccination movement, and anxiety among media and public health professionals about the influence of high-profile vaccine skeptics like Jenny McCarthy suggests that popular celebrities and alternative healthcare providers (e.g. naturopaths, chiropractors) may be the source of rising concern among parents about vaccine safety.

Our results show that the three *most trusted* sources for health information overall are physicians (89 percent trust, 7 percent do not trust), public health officials (83 percent trust, 12 percent do not trust), and academics (77 percent trust, 15 percent do not trust). By contrast, the *least trusted* sources are popular celebrities such as Gwyneth Paltrow and Oprah Winfrey (8 percent trust, 82 percent do not trust). Celebrity physicians such as Dr. Oz have higher levels of trust than other celebrities do (25 percent trust, 59 percent do not trust), but overall remain strongly distrusted in comparison with most other sources. This should also be a point of concern given the tendency of these sources to pedal junk science and the fears that these types of sources will influence medical decision-making 12 ^,^ 13. Finally, roughly equal numbers of parents expressed trust and distrust in the news media (46 trust, 47 do not trust). This finding is intriguing given how many of our respondents stated they would rely on media outlets as their main source of information during an outbreak event 14.

Trust in institutional sources is polarized when we compare parents who vaccinate their children and those who do not. Not surprisingly, levels of trust in physicians, public health authorities and academics were much higher among vaccinators than non-vaccinators: approximately 90 percent of parents whose children have been vaccinated agreed that these sources can be “trusted to do what is right” compared to only 6 percent who disagreed. Among non-vaccinators, a much smaller group overall, 55 percent expressed trust in these sources compared to 37 percent who did not. Vaccination status was also significant when considering levels of trust in the pharmaceutical industry. While drug industry distrust was generally high with both groups, it was significantly higher among non-vaccinators (80 percent compared to 51 percent). And in the case of celebrities, despite the concerns of public health authorities that parents will blindly follow the advice of celebrity anti-vaxxers like Jenny McCarthy, there was no significant difference between vaccinators and non-vaccinators: both groups agree that popular celebrities and medical celebrities are not trustworthy sources of vaccine information.

*Vaccine Hesitancy: What’s to be done?*

Health officials have used numerous approaches to persuade parents who do not vaccinate their children to change their views and behaviours and to reinforce positive vaccine behaviour among those who already do. Attempts to frighten parents about the risks of disease or correct false claims about vaccines have been largely ineffective, and may be counterproductive 15. Sandman and Lanard^16^ argue that there is a fine line between warning the public that a given risk may be potentially worrisome without actually scaring people. Yet, officials often resort to scare tactics and have occasion to use dramatic and vivid imagery to frighten or shame parents into having their children vaccinated. Images of sick children may effectively provoke fear, worry, and other emotions that can be persuasive. Yet, the use of emotionally evocative images may also strengthen beliefs in a vaccine/autism link among a core group of parents, while dramatic narratives that describe the risks to infants of under-immunization can increase self-reported beliefs about serious side effects of vaccines 15.

While our study did not involve experimental testing of risk communication interventions, we did ask participating parents to reflect on messages that public health officials often use to persuade those who are vaccine hesitant, and to indicate which, if any, they feel work best at increasing vaccine uptake among this group (Table 1).

The majority of respondents believed that all of the suggested messages, with the exception of shaming, are likely to be effective in persuading parents to have their children vaccinated. Interestingly, while almost two-thirds of our parents agreed with the statement, “parents who do not have their children immunized (except in cases involving medical exemptions) are irresponsible,” nearly as many (64 percent) believed that this message would be unlikely to change the immunization behaviours of other parents. The relationship between these two questions was moderate and statistically significant: parents who strongly disagree that those who do not vaccinate their children are irresponsible are more likely to dispute the efficacy of shame-based messaging to change vaccine hesitant behaviour. However, parents who do vaccinate their children also believe that this approach is unlikely to be effective in changing behaviour.

The messages that respondents overall felt would most likely work with vaccine hesitant parents were those that emphasized the scientific evidence showing that vaccines are safe and effective (47 percent), followed by messages about the likelihood of catching a serious childhood illness without vaccine protection (40 percent) and those which vividly detail the effects of childhood diseases (37 percent). Among the small number of parents who self-identified as holding anti-vaccine beliefs, the only message that showed any hope of effectively persuading parents like them was, “provide positive encouragement and emphasize that vaccines are strongly recommended, but ultimately the decision is theirs to make” (77 percent). All other messages generated very strong negative reactions for non-vaccinating parents, indicating they would all be unlikely to ameliorate their hesitant beliefs and behaviours.

Finally, research participants were invited to suggest other possible risk communication approaches for persuading vaccine hesitant parents to change their beliefs and behaviours. We coded the 857 discrete responses to this question into numerous other categories, of which the most commonly cited recommendation (28 percent of respondents) was, “use of research to debunk vaccine myths.” Among parents with strongly vaccine hesitant views, messages based on “showing compassion” and “communicating honestly about risk” were most common, although this represents a very small baseline of responses.

The results of our research illustrate a potential disconnect between what parents of young children believe will be effective in persuading parents who are vaccine hesitant with what the available experimental research already tells us: more evidence, statistics and debunking strategies are the least likely to work 15 ^,^ 17 ^,^ 18. Of concern, all of these approaches have been shown in other research to have little to no positive impact on vaccine uptake, and may be counterproductive. If it is assumed that parents who do not have their children vaccinated do so because they lack appropriate knowledge and information, or because they have been duped by anti-vaccine celebrities and activists, then perhaps it is not surprising that parents (and health professionals) would try to address that problem with more science, data and evidence.

The responses about vaccine risk messaging from parents who hold more strident vaccine-hesitant views are worth considering to the extent they reflect the value and importance of expressing empathy and compassion, and providing support and positive encouragement as a means for building trust with parents over the longer term, even if in the short term that does not lead to changes in immunization behaviour. Openness, dialogue, empathy, and respect are foundational values to ethical and effective risk communication 19 ^,^ 20 ^,^ 21. And while they might not yield an immediate shift in vaccination behaviours, they may, over the longer term, be our most effective protection against the wicked problem of vaccine hesitancy.

**Table 1:** Risk Messaging to increase vaccine uptake

	Very Likely	Somewhat Likely	Not Very Likely	Not At All Likely	N=
a. Messages should emphasize scientific evidence showing that vaccines have a strong record of safety and effectiveness at reducing serious childhood illnesses.	469 (47.0%)	411 (41.2%)	94 (9.4%)	24 (2.4%)	998
b. Messages should emphasize the statistical likelihood of catching a serious childhood illness like measles or whooping cough without being vaccinated.	401 (40.1%)	449 (44.9%)	117 (11.7%)	33 (3.3%)	1000
c. Messages should use shaming techniques to persuade parents they have a moral duty to vaccinate their children and protect their community.	112 (11.3%)	248 (24.9%)	313 (31.5%)	322 (32.4%)	995
d. Messages should vividly detail the negative effects of childhood diseases, for example photographs of seriously ill children and the consequences for their parents.	365 (36.5%)	440 (44.0%)	145 (14.5%)	50 (5.0%)	1000
e. Messages should provide positive encouragement to parents and emphasize that vaccines are strongly recommended, but ultimately the decision to vaccinate their children is their choice to make.	294 (29.4%)	418 (41.8%)	212 (21.2%)	76 (7.6%)	1000

## Limitations and Conclusions

There are some limitations to this study worth noting. First, although the online panel used for our survey is constructed to be representative of the Canadian population in terms of age, region of residence, income and education, selection bias and non-response bias cannot be ruled out. However, the sociodemographic characteristics of our respondents are not significantly different from those of the Canadian population of parents with children aged 5 and younger. Second, the MMR vaccination decision for the child was self-reported by parents which could lead to recall bias, and there was no other measure within the study to assess parental vaccine hesitancy attitudes along a broader spectrum. Hence, as most respondents reported that their child was vaccinated, their reflections on the standard communication messages used by public health to persuade parents about the benefits of vaccination, as well as those suggestions provided by parents that could be persuasive in encouraging parents to vaccinate their children, cannot be expected as being effective specifically for vaccine hesitant parents. Relatedly, messaging deemed to be more acceptable by anti-vaccination parents – namely, public health messaging that both strongly recommends childhood vaccinations while equally expressing empathy and compassion for parental choice – needs further empirical testing, either in an experimental design or through intensive qualitative research.

Despite these limitations, our findings make important contributions to our understanding of vaccine hesitancy and the communication challenges it presents. The data illustrate a combination of positive and troubling news for health communicators who are struggling to find the risk communication comfort zone that builds trust with hesitant parents while increasing vaccination rates. First, our survey indicates that a strong majority of Canadian parents consider vaccines safe and effective, and trust doctors and public health officials to provide timely and credible vaccine-related information, while expressing much lower trust in politicians and industry sources. At the same time, indicators of vaccine hesitancy are prevalent: a significant number of parents either believe or are uncertain about the scientifically unproven link between vaccines and autism, and a concerning number of them worry that vaccines could seriously harm their children.

Despite the widely held belief that social media are replacing legacy news organizations as a major source of health news and information, and thus present a potential threat to public health communication as a site of 'fake news' and disinformation, most parents of young children still rely on traditional media and official government websites for timely and credible information about vaccines. This is particularly true during outbreaks of vaccine preventable disease—there are clearly important opportunities to use these extraordinary moments to reinforce positive messaging about the benefits of vaccination. Our data also show that parents place a high level of trust in government websites and expect or assume those sites to be regularly updated with reliable and timely information. Medical and public health officials working in government departments and agencies should pay special attention to this finding.

Finally, parents report high levels of support for pro-vaccine messaging that emphasizes the science of vaccine safety and effectiveness, vividly depicts the consequences of disease, and debunks the myths and misinformation about vaccination. Future research should build on these results to test risk communication interventions with parents who occupy different standpoints along the vaccine hesitancy spectrum.

Taken together, these results contribute to a growing body of research on vaccine hesitancy and health risk communication, and are highly relevant in a context where health officials continue to struggle to strengthen rates of community protection and inspire confidence about the benefits of vaccines for protecting public health.

## Competing Interests

The authors have declared that no competing interests exist.

## Data Availability

Data are available from the figshare repository at the following URL: https://figshare.com/s/055c898abb39690b0871.

## Corresponding Author

Josh Greenberg: joshuagreenberg@cunet.carleton.ca
